# Health care providers’ and persons with disabilities’ recommendations for improving access to primary health care services in rural northern Ghana: A qualitative study

**DOI:** 10.1371/journal.pone.0274163

**Published:** 2022-09-16

**Authors:** Ebenezer Dassah, Heather M. Aldersey, Mary Ann McColl, Colleen Davison

**Affiliations:** 1 School of Public Health, Kwame Nkrumah University of Science and Technology, Kumasi, Ghana; 2 School of Rehabilitation Therapy, Queen’s University, Kingston, Ontario, Canada; 3 Department of Public Health Sciences, Queen’s University, Kingston, Ontario, Canada; PLOS: Public Library of Science, UNITED KINGDOM

## Abstract

In Ghana, many persons with physical disabilities are members of populations who face health disparities including physical, structural, knowledge, attitudinal and financial barriers to various health care services compared to those without disabilities. However, there is limited evidence on how to improve access to primary health care services for persons with physical disabilities. This study aimed to understand persons with physical disabilities’ experiences and health care providers’ perspectives for improving access to primary health care for persons with disabilities in rural Ghana. We used a qualitative approach and interviewed 33 persons with physical disabilities and health care providers, and thematically analysed data from in-depth interviews. We identified 4 major themes. According to the participants, health care could be more accessible by: i) Making it more affordable; ii) Increasing the availability of providers and services; iii) Providing more education about system navigation; and iv) Improving access to disability friendly health facilities and equipment. Participants’ recommendations were nested in the areas of policy and practice modifications. Policy makers need to consider supporting persons with physical disabilities who cannot afford non-medical services (i.e., cost of transportation). In terms of practice, the provision of education and training related to physical disability issues should be extended to both clinical and nonclinical health workers for better client centered care. There is an urgent need for policy makers and relevant key stakeholders to include persons with physical disabilities in designing and implementing policies and programs to ensure that they are meeting their needs.

## Introduction

Article 25 of the Convention on the Rights of Persons with Disabilities recognizes that persons with disabilities have the right to the highest attainable standard of health without discrimination on the basis of disability [1]. Increasing evidence, however, indicates that persons with disabilities experience physical, structural, knowledge, attitudinal and financial barriers to various health care services [2–4]. This is even more exacerbated among those with physical disabilities who are at higher risk of not taking up or delaying care, and also having unmet medical care needs compared to those without disabilities [4, 5]. Persons with physical disabilities (i.e., those with self-reported physical limitations or limitations in activities of daily living, including standing, walking, climbing stairs, bending, reaching and grasping) commonly exhibit health behaviors that include unhealthy eating and sedentary lifestyles [6, 7]. As such access to appropriate health care services is critical to achieving optimal health outcomes for persons with physical disabilities [8]. The lack of timely access to health care could result in the development of chronic and secondary conditions as well as the exacerbation of disabling conditions, and thus resulting in poorer health outcomes for persons with disabilities [2, 4]. Given these underlying causes of disparities, it is critical to develop and implement strategies to improve access to quality health care access for individuals with disabilities. This is especially important, as researchers suggest that identifying strategies that integrate the needs of persons with disabilities in primary health care (PHC) systems could help address health disparities, and therefore, should be the leading research priority in low- and middle-income countries (LMICs) [9, 10].

Within the last two decades, Ghana has made policy commitments to address the health of individuals with disabilities. For instance, the government passed a Disability Act that has many provisions related to reducing health care disparities, including free medical and rehabilitation services and disability friendly health care facilities [11]. Similarly, the government introduced a national health insurance scheme as a health policy to make health care affordable thus increasing access to basic health care for all residents, including those with disabilities [12]. These policies, however, have not been translated into practice as growing evidence suggests that interactions between persons with disabilities and the health care system often culminate in access barriers such as lack of necessary services, financial constraints, inaccessible health care facilities and equipment, and negative attitudes from providers [12–15].

Previous research on disability and health in the Ghanaian context has contributed to knowledge on factors that facilitate or impede persons with disabilities’ access to health care services [12–15]. These studies focused largely on either the perspectives of health care providers or persons with disabilities in general. The evidence has also drawn inferences for improvement in health care provision for the general disability population in Ghana.

Consequently, there is limited research that has explicitly focused on recommendations of both service providers (i.e., health care providers) and service users (i.e., persons with disabilities) to improve health care delivery for persons with disabilities [16]. Therefore, this study aimed to understand recommendations from health care providers and persons with physical disabilities to improve health care delivery for persons with disabilities in Ghana. Specifically, we sought to answer the following research question: what are providers’ and persons with physical disabilities’ recommendations on improving access to PHC services for persons with physical disabilities in rural Northern Ghana? Deepening our knowledge about both service users’ and providers’ recommendations may be influential in addressing health disparities and improving the quality of health care delivery for persons with disabilities in Ghana and similar LMICs contexts. This will, in turn, contribute to the realization of Sustainable Development Goal 3 that is anchored on universal health coverage, access to quality health and equity in health care as crucial for sustainable development [17].

### Overview of the Ghana’s health care system and primary health care delivery

Ghana has a comprehensive, pluralistic and multifaceted health care delivery system that comprises public, private for-profit, private not-for-profit and faith-based healing systems [18]. The public or government-run health care sector largely provides health care services to citizens, and it is mostly administered and implemented by the Ministry of Health (MOH) and the Ghana Health Services (GHS) respectively [19]. Specifically, the MOH coordinates the implementation of health policies with other relevant ministerial agencies, whereas the GHS is responsible for health service delivery. Health care services are delivered at three main levels: primary, secondary, and tertiary levels. The primary level is made of health facilities such as Community-based Health Planning and Services (CHPS) compounds, clinics, health centers, polyclinics and district hospitals. The secondary level comprises facilities at the regional level such as regional hospitals. The tertiary level is composed of the tertiary hospitals such as the teaching hospitals that are mostly connected with universities and take referrals for advanced and specialized care [19].

This study focuses on the primary level, as all the PHC services in rural Ghana are provided at this level. In terms of hierarchical organization of this level, the CHPS compounds are the most basic PHC facilities that provide basic medical services and conduct health promotion in many rural communities in Ghana. These CHPS compounds primarily have community health officers/community health nurses and community health volunteers as the main health care providers. The clinics and health centers are mostly staffed with a physician assistant or nurses as well as specialists in the fields of midwifery, laboratory services, public health, environmental health, and nutrition. These facilities provide both preventive and curative services, and to some extent minor surgical services. The polyclinics and district hospitals deliver comprehensive services that include preventive and curative care, outpatient and in-patient services, and health promotion. These facilities comprise a physician(s), physician assistants, nurses, pharmacists, laboratory technicians, auxiliary nurses and other support personnel.

### Ghana’s National Health Insurance Scheme

The government of Ghana adopted a National Health Insurance Scheme (NHIS) in 2003, and implemented as national program in 2005, as a pro-poor health policy to promote universal coverage and equity in access to health care services [20, 21]. The NHIS is a strategy for financing health care with the ultimate goal of providing universal health coverage for all Ghanaians, irrespective of their socio-economic background [20, 22, 23]. The operations of the NHIS is detailed in a plethora of studies [20–23]. In brief, individual must pay an annual subscription fee of 25 Ghana Cedis (approximately 4.50 US Dollars as at March 2021) in order to be enrolled for the coverage of health care services. Premium exceptions are, however, provided for persons with disabilities that are categorized as indigent (i.e., those who extremely poor and marginalized within criteria set out in a livelihood empowerment against poverty program). Nevertheless, it is difficult to identify the indigent because there is a lack of clarity about determining poverty in Ghana [21]. Given this, persons with disabilities continue to experience financial difficulties in accessing health care services despite the existence of the NHIS [12].

### Theoretical framework

We employed access to health care framework to understand the topic. The concept of access to health care remains a nebulous and obscure to consumers, health care providers, and policymakers [24]. Given this, numerous variations persist in the conceptualization of the concept [25, 26]. Nevertheless, an earlier definition of access to health care was primarily centered on the use of services or realized access [27, 28]. The actual use of services was supported by Donabedian [29] who posited that proof of access required use of service, not merely presence of a facility. Hence, the use of services is determined by predisposing (characteristics of the individual, i.e., age, gender, race), enabling (system or structural factors, i.e., income, health insurance status), and need (severity of illness) factors [28]. These individual and systemic characteristics was further expanded by researchers who conceptualized access as a “fit” between the client/individual (demand) and health care system (supply) across different dimensions, including availability, geography, affordability, accommodation, timeliness, acceptability and awareness [25, 30–33]. Thus, in this study, we conceptualized access to health care as the “fit” of how well the health care system/providers (i.e., health care providers) meet the needs of their clients (i.e., persons with physical disabilities).

## Materials and methods

### Study design

This article forms part of a larger doctoral study designed to understand disability and access to PHC services in rural areas. Some study findings have been reported elsewhere [10, 14, 15]. However, this article focuses on a dominant theme that emerged from the qualitative data analysis. We followed a qualitative descriptive design to address the study objective. We chose qualitative descriptive design, as it affords researchers with the flexibility to understand human experiences, and also aims to provide practical answers that are useful to health care practitioners and policy makers [34]. We collected data between January and March 2017.

### Ethics

The study was approved by the Queen’s University Health Sciences and Affiliated Teaching Hospitals Research Ethics Board in Canada and the Upper West Regional Administration of the Ghana Health Services. We obtained signature or thumbprint from each individual participant to indicate consent to participate in the study and to have their data included in subsequent publications. We gave participants refreshments in acknowledgement of the time they gave to participate in the study.

### Research settings

This study was conducted in the upper west region of Ghana. The region is one of the 5 regions that constitute northern Ghana. It has a total population of 702,100 (2.8% of Ghana’s population), rural population rate (83.7%) and rate of persons with disabilities (3.7%) [35]. The region has one of the highest poverty incidences; approximately 71% of the population live below the annual poverty line of 1314 Ghana Cedis (328.50 US Dollars) [36]. Further, human resources including physicians, nurses and specialized service providers are lacking in the region. This situation combined with limited health care facilities and a poor road network further compound access to health care services when needed [19]. The region currently has 11 administrative assemblies—5 municipal and 6 district assemblies. We conducted the research in local communities in one municipal (Jirapa) and three district (Daffiama/Bussie/Issa/, Nadowli/Kaleo and Wa West) assemblies. We selected these assemblies because of the first author’s familiarity with the socio-cultural context (i.e., cultural practices, values, and language) which are important in obtaining rich information [37]

### Sampling and recruitment of participants

The detailed sampling and recruitment procedures for participants with physical disabilities are explained elsewhere [15]. In summary, we used a purposive sampling technique to achieve maximum variation in the demographic characteristics of participants with physical disabilities (e.g., age, gender, education, location and level of social support). Inclusion criteria were: (a) adults (18–64 years) with any form of physical disability, (b) ability to communicate in English and/or Dagaare (the widely spoken language in the study areas) and (c) willingness to participate. The first author recruited potential participants through the heads of disabled people’s organizations (i.e., the Ghana Society of the Physically Disabled) in the study areas. Specifically, the heads of the organizations circulated detailed information about the study to their members, thus providing access to potential participants. The first author then recruited participants that met the eligibility criteria. Additionally, we purposively sampled health care providers based on the following inclusion criteria: (a) provision of health services to persons with physical disabilities in their health facilities; (b) ability to communicate in English and/or Dagaare; and (c) willingness to participate. We sampled these participants based on age, gender, specialization, district, type of facility working, and number of years working in the profession to achieve diverse perspectives. To commence recruitment of eligible participants, we first obtained permission from the Upper West Regional Administration of Ghana Health Services. The first author then identified and recruited participants that met the inclusion criteria.

In total, 33 participants (comprising 18 persons with physical disabilities and 15 healthcare providers) participated in the study. See Tables 1 and 2 for some of the characteristics of participants with disabilities and health care providers respectively. We determined the sample size using the concept of ‘information power’ wherein an adequate sample size is defined in terms of study aim, sample specificity, use of established theory, quality of dialogue, and analysis strategy [38].

**Table 1:** Demographics of persons with physical disabilities.

ID	Gender (M/F)	Mobility Device	Level of Education	Social Support
1	F	Tricycle	Secondary	Lives with family
2	F	Walking stick	None	Lives alone
3	M	Tricycle	Primary	Lives alone
4	F	No device	None	Lives alone
5	M	Tricycle	None	Lives with family
6	F	Tricycle	Secondary	Lives with family
7	M	Tricycle	Tertiary	Lives with family
8	F	No device	None	Lives with family
9	M	Powered	Tertiary	Lives with family
10	M	Tricycle	None	Lives with family
11	F	No device	None	Lives alone
12	F	Tricycle	Tertiary	Lives with family
13	M	Tricycle	Secondary	Lives with family
14	M	Tricycle	Primary	Lives with family
15	F	Walking stick	Primary	Lives with family
16	F	Tricycle	None	Lives alone
17	M	Tricycle	Primary	Lives with family
18	M	Walking stick	None	Lives with family

**Table 2:** Demographics of health care providers.

ID	Gender (M/F)	Specialization	Type of facility	Years in profession
1	F	Community health nurse	CHPS compound	5
2	F	Physician assistant	Health centre	22
3	M	General nurse	Health centre	12
4	F	General nurse	Health centre	21
5	M	Community health nurse	CHPS compound	2
6	F	Physician assistant	Hospital	15
7	M	General nurse	Health centre	1
8	F	General nurse	Hospital	31
9	M	Community health nurse	CHPS compound	3
10	M	Physician assistant	Health centre	18
11	F	General nurse	Health centre	12
12	F	Auxiliary nurse	CHPS compound	4
13	M	Physician assistant	Health centre	32
14	M	Physician	Hospital	16
15	F	General nurse	Health centre	25

### Data collection

The first author conducted 33 in-depth interviews (16 in Dagaare, 12 in English, and 5 in both Dagaare and English) in various locations including, disability resource centers, participants’ homes and health facilities, and at a time convenient to each participant and the researcher. Interview questions for all the participants centered on access barriers and the ways of improving health care and assisting persons with disabilities to meet their health care needs at the PHC level. Each interview was digitally recorded with the consent of the participants. Field notes were taken during and after each interview recording non-verbal cues and researcher reflections. The interviewer repeatedly used prompts to elicit detailed information or clarification of certain concepts used by the participants. The interviews lasted between 45–60 minutes.

### Data management, analysis and trustworthiness

After the interviews, the first author also simultaneously translated and transcribed the Dagaare interviews in English. Two independent researchers fluent in both English and Dagaare cross checked all the transcribed and translated interview transcripts (See Dassah et al. [15] for a detailed information about these processes). We subsequently analyzed the interview transcripts using thematic analysis, which included; (a) familiarizing oneself with the data, (b) generating initial codes, (c) organizing the codes and searching for themes, (d) reviewing and refining the themes, and (e) writing a report [39]. We also established trustworthiness using other strategies that included consistent use of debriefing sessions, in-person meetings, reflexivity and consensus on the findings [40, 41]. We used NVivo software to manage and store the data [42].

## Results

We identified 4 major themes. According to participants, health care could be more accessible by: i) Making it more affordable; ii) Increasing the availability of providers and services; iii) Providing more education about system navigation; and iv) Improving access to disability friendly health facilities and equipment. We provide details of these themes and supportive quotations from the participants in the section below.

### Making health services more affordable

All the participants with disabilities highlighted that health services need to be affordable. They especially remarked that the national health insurance program is a good social safety net that enables access to health care. This is because the health insurance offers free services to persons who experience marginalization and covers almost all the health conditions that affect rural residents, including those with disabilities. Nevertheless, persons with physical disabilities explained that the health insurance scheme needs reorganization to make health services more affordable. They also noted that reorganization could enhance timely access to services that might reduce the exacerbation of their physical conditions. Ensuring that health insurance covers the indirect costs of services was also a common recommendation by the participants. In particular, persons physical with disabilities discussed that indirect costs were related to paying for their own transportation and that of their family members escorting them to health facilities. Beyond transportation, indirect costs included buying food and other personal items while seeking care. In some cases, indirect costs included opportunity costs associated with family members taking time off work in order to accompany persons with physical disabilities accessing health care services. One participant suggested a need for the health insurance scheme to cover indirect costs like ambulance services.

*It will be nice if the health insurance could cover transport services*, *like ambulance services so that when we are referred to other facilities*, *they don’t ask us to pay for the service*. *It will help us [persons with disabilities] a lot in reducing the financial burden of accessing health services*. (Physical Disability, Male)

Most of the providers also echoed similar sentiments indicating that the reorganization of the health insurance would enhance quality and timely health care delivery. One provider remarked:

*If we look at the health insurance critically*, *you can see that it needs serious reorganization*. *For instance*, *in most cases*, *the health insurance scheme owes service providers*. *As such there should be regular payment to providers offering health care service to our facilities for quality and timely care delivery to persons with severe conditions*. (Physician Assistant, Female)

### Increasing the availability of providers and services

Participants with disabilities identified the availability of providers and services as a key recommendation. They particularly suggested that the recruitment of allied health professionals (i.e., physiotherapists) would help improve service delivery to persons with physical disabilities. For instance, they indicated that the presence of rehabilitation specialists is necessary, as they can understand their conditions better and provide care that meets their specific and unique needs. Given this, they expressed an urgent need for rehabilitation professionals. A participant living with a disability highlighted:

*We really need disability specialists here [name of community] to handle our health care needs*. *It is something we usually complain [about] but don’t know how to fight for it*. *People in authority really need to look at this problem and find a solution for us*. (Physical Disability, Female)

The participants also called for closer proximity of health facilities to their communities. Providers echoed similar views, emphasizing that having health care facilities closer to communities would ease the burden of persons with physical disabilities having to navigate unfriendly built environments in order to receive health care outside of their communities. Providers also shared that they would like to have regular supply of medical equipment and supplies to provide the needed services for persons with physical disabilities. Some participants also suggested that extending mobile vans or clinics with rehabilitation specialists could encourage greater service provision to persons with physical disabilities in rural communities. A provider described:

*Since there is always lack of disability specialists in most rural facilities*, *I think it might be wise to make mobile clinics and specialists come here maybe on biweekly or monthly basis to provide care*. (Community Health Nurse, Male)

### Providing more education about system navigation

A notable strategy for improving access to PHC as suggested by participants with physical disabilities was to incorporate disability issues in health-related training institutions, and also provide regular training to providers. A majority of persons with physical disabilities stressed the importance of education and training as a tool in understanding the needs of persons with physical disabilities. A participant living with a disability stated:

*I think health workers really need lot of training to understand our needs*. *Sometimes these health workers just shout on us as if we are not human beings*. *Some are really rude*, *insensitive and above all not well trained on how to communicate with us*. *I think this is the time to invest in their training to understand disability issues*. (Physical Disability, Male)

Some providers also echoed similar views reaffirming that continuing professional development training seminars on disability issues should be extended to frontline health care providers, irrespective of their previous education and training. They remarked that such trainings could help them update their knowledge and skills on disability conditions. The providers further observed that non-medical staff such as administrators, cleaners and accountants are often not involved in education and training programs. Thus, providers recommended that such trainings should involve these non-clinical staff.

*Like people think it is only nurses and physicians that need training to better serve persons with disabilities*. *We actually need to extend regular trainings to every worker in health care facilities*. *This is because there is the possibility that every health worker will have an encounter with persons with disabilities in our facilities*. (General Nurse, Female)

Additionally, adopting positive attitudes and raising awareness about disability issues among the general public was also highlighted by the participants with physical disabilities as key in improving access to PHC services. Given the stigmatization and discrimination faced by persons with physical disabilities, participants recommended that positive attitudes from the public and the health system are necessary to ensure appropriate access to health care. One participant with a disability said such positive attitudes could be in the form of giving persons with physical disabilities priority in queues for health care services, especially those that cannot join in a queue for long hours due to their physical situation. In terms of awareness creation, participants commented on the need to adopt public education programs about disability at the community level and also mainstream disability issues into health policies and programs. Participants noted that such strategies could change society’s negative views about disability and support inclusive health care services in rural communities.

### Improving access to disability friendly health facilities and equipment

Participants with physical disabilities noted that most of the health facilities need improvement to make them disability friendly. The participants particularly highlighted that attention should be devoted to redesigning or constructing new health facilities to accommodate their needs. One participant with a physical disability suggested how this could be achieved:

*I believe all the stakeholders involved in disability issues need to work hand in hand to ensure that the current facilities or those yet to be constructed are disability friendly*. *For instance*, *building regulators*, *architects and the health ministry authorities need to work together to achieve disability friendly health facilities*. (Physical Disability, Female)

Although much of the conversation was focused on the health facilities, it also extended to disability friendly medical equipment (e.g., weighing scales, hospital beds and chairs) and spaces. There were differences in opinion between providers and persons with physical disabilities on how the provision of medical equipment could be done. For instance, persons with physical disabilities recommended the need to replace all existing equipment with more disability friendly ones within the shortest possible time, whereas providers described the provision of disability friendly medical equipment as the realistic short-term goal, while advocating for improvement in the facilities. One health care provider noted:

*To me redesigning health facilities should be our long-term goal given the limited financial resources we receive from the government*. *For now*, *let’s all focus on how we can get medical equipment that can serve the needs of persons with disabilities*. (Physician, Male)

Regardless of their differences, the consensus was that there was a lack of disability friendly medical equipment in health care facilities. Given this, participants urged authorities in the health sector to factor in the needs of persons with physical disabilities when procuring medical equipment and supplies. It was evident from the interviews that lack of disability friendly health facilities and equipment often deters persons with disabilities from seeking health care services when needed. Importantly, participants’ recommendations point to a need for monitoring of the implementation of the provision of the disability policy that commits to making public spaces and facilities disability friendly. Such disability friendly public space is supposed to include the presence of ramps and large washrooms and adequate space within providers’ offices for wheelchair users.

## Discussion

This study sought to understand recommendations from health care providers and persons with physical disabilities to improve health care delivery for persons with disabilities. We identified four main themes that both health care providers and persons with physical disabilities considered necessary to improve health care access for persons physical with disabilities. Overall, the study highlights a discrepancy between participants’ perspectives, experiences and remaining recommendations and statements in various policy documents supporting access to health care services for persons with disabilities in Ghana. For example, the Ghana’s Disability Act states that accessible buildings and other infrastructure must be provided [11]; however, this is not reflected in reality, as participants in this study still recommended the need for disability friendly health facilities and medical equipment.

Making health care more financially accessible was a commonly reported recommendation in this study. Given that health insurance schemes can result in positive health outcomes for persons with disabilities [15, 43], participants advised the government to strengthen the health insurance scheme by making regular payments to service providers to improve access. This suggestion is crucial for persons with disabilities, as they are less likely to afford health care services or join work-related health insurance schemes, resulting in delaying or forgoing health care services when needed [44]. Additionally, the indirect cost of health care services is a key health care expenditure, and participants in this study indicated the need for health insurance to cover such cost. This supports literature indicating that persons with disabilities face extra costs associated with accessing primary health care services, including accessible transportation, meals and opportunity costs of accompanied family members [15, 43]. These issues highlight that the mere presence of national policies and programs to overcome financial barriers experienced by persons with disabilities do not, in and of themselves, guarantee access to health care. Not surprisingly, consistent evidence indicates that persons with disabilities tend to experience poorer health outcomes, have higher health care costs and are 50% more likely to experience catastrophic health care expenditures than their counterparts without disabilities [44, 45]. Therefore, the achievement of universal health coverage can be necessary when attention is given to making health care services affordable for all [43, 46].

Remarkably, given the challenges of the health insurance, interventions that are geared towards addressing poverty and raising the standard of living for persons with disabilities are paramount in ensuring access to health care. This is particularly essential for those with physical disabilities who often experience financial challenges due to limited employment opportunities when living with disabilities in Ghana [47]. Providing employment opportunities could be a source of empowerment that can enable them to meet the direct and indirect costs of health services. As such, the government should prioritize hiring persons with disabilities in State-run agencies, for instance. Additionally, the government should ideally strengthen the implementation of social interventions such as the Livelihood Empowerment Against Poverty program that provides cash and free health insurance to extremely poor people, including people with severe physical disability without any productive capacity [48].

An overarching theme in this current study was participants’ recommendations for the improved availability of services and providers in rural areas to enhance access to PHC services. This is in line with existing evidence reporting that the availability of professionals and facilities in rural areas has the potential to increase early access to PHC services [10] and subsequently reduce the risks of secondary health conditions associated with physical disability [49]. Crucially, the recent Declaration of Astana has recognized the importance of mainstreaming rehabilitation services into PHC systems, as it can help achieve the goal of universal health coverage [50]. Participants in this study also highlighted a need for the availability of rehabilitation professionals and services in rural health facilities. However, with the lack of opportunities and incentives, people in the northern parts of Ghana are witnessing shortage of human resources in health care [51]. This deficit is likely to be exacerbated for persons with disabilities who may need the services of rehabilitation professionals. For instance, Ghana has a total of 160 registered physiotherapists (as of December 2018) serving over 30million people [52], and the first group of locally trained occupational therapists only graduated in 2017. In view of this, there is a need to train more rehabilitation professionals to serve in rural areas. To achieve this, admissions and funding packages with service agreements could be rolled out to prioritize persons willing to start their employment in rural areas. Evidence has also shown that monetary and non-monetary incentives (i.e., policy regulations, educational opportunities, professional support, improved living conditions, health facilities and personal transportation) are key motivators in attracting and retaining health workers, including therapists, in rural areas [53, 54].

The findings from this present study and others conducted elsewhere [13, 16] indicate that education and awareness creation can boost persons with disabilities’ access to PHC services. Evidence shows that the pathway to health care services for persons with physical disabilities in Ghana is often impeded by stigmatization and discrimination [15]. This situation can make persons with disabilities feel frustrated and may leave them unwilling to engage with the health care system at all [13]. Unsurprisingly, persons with disabilities in this study suggested the need for education and training of providers around how to interact with them. Of particular importance are responses from frontline health care providers advocating the need to train non-clinical staff on disability issues. We argue that such training should form an integral part of requirements prior to working in health care facilities. Generally, education and training for all health staff on disability issues should include persons with disabilities to help health workers understand their perspectives and experiences living with disability, as it is paramount to enhance the latter’s access to health care services [55].

Further, participants attested that the provision of disability friendly health facilities and equipment will facilitate inclusive and accessible health care for all. Consistent evidence shows that physical inaccessibility of health facilities and medical equipment is a major logistical challenge faced by persons with physical disabilities globally. For instance, evidence revealed that persons with physical disabilities encounter significant access barriers, including entering and navigating health care facilities and using examination equipment (i.e., tables and chairs) [8, 56]. Persons with disabilities often dealt with these barriers by asking others for assistance—a situation that sometimes made them feel vulnerable and embarrassed. These accommodation difficulties reveal that the mere presence of health facilities and equipment does not guarantee access, unless persons with disabilities are able to use them [15, 57]. Thus, access depends on the complex interactions between the individual circumstances and the built environment that ultimately determine whether health services can accommodate the needs and wants of persons with disabilities.

Finally, our findings suggest that the improvement of health care services for persons with physical disabilities will require interventions at the supply side (i.e., from the health care system/services) and the demand side (i.e., from persons with physical disabilities) to effectively meet the needs of persons with physical disabilities. This suggestion aligns with the conceptualization of access that reflects a good fit between supply of, and demand for, health care services [25, 30, 31]. In this study, the findings illustrated a discrepancy between demand and supply. For instance, most suggestions offered by the participants point to the need to address health system characteristics, indicating an unfavorable fit between the characteristic of the health care system and that of persons with physical disabilities. In order to effectively address the needs of persons with physical disabilities, it is necessary focus on both demand and supply factors simultaneously.

### Limitations and recommendations

Despite the many strengths of this research, there are limitations that should be acknowledged when interpreting the findings. First, although participants have provided insights into improving access to PHC, it is not clear whether these recommendations would lead to improved access for persons with physical disabilities in rural areas. Thus, the findings of this study reinforce the call for a more nuanced research to assess whether the recommendations made by health care providers and persons with disabilities can effectively improve access to and provision of quality health care [16]. Second, while we explored the study from the perspectives of both providers and service users to provide a comprehensive understanding of the topic, these participants were not involved in the data analysis. There is the possibility that their involvement in the whole research process could have provided additional unique insights. We therefore argue that future research could employ more participatory research approaches such photographs (e.g., photovoice). Third, the study involves a few selected districts and municipal assemblies in only one region in northern Ghana. Thus, the findings can be understood as specific to participants in this setting. Nevertheless, it would be useful to conduct quantitative research in other geographical regions to get a broader idea of these recommendations in the country. Finally, as we did not disaggregate the data by gender, there is a need for further research on how gender might influence the recommendations by the participants. This is particularly necessary given the recent United Nations call for gender mainstreaming in disability issues as a major pillar in achieving the 2030 Agenda for Sustainable Development [17, 58].

## Conclusion

Participants believe that improvement of PHC services should focus on making health care services readily affordable, available, acceptable and accommodate the unique and specific needs of persons with disabilities. These recommendations were nested in the areas of policy and practice modifications and can serve as a guide to boosting access to PHC services for persons with disabilities. In particular, policy makers need to consider the expansion of health policies to support persons with disabilities who cannot afford non-medical services (i.e., cost of transportation). In terms of practice, the provision of education and training on disability issues should be extended to both clinical and nonclinical health workers to improve client centered care. We also conclude with a call for policy makers and relevant key stakeholders involved in disability and health to include persons with disabilities in designing and implementing policies and programs to ensure that they are meeting the needs of persons with disabilities. This aligns with the Disability Movement empowerment slogan of *“Nothing about us*, *without us”* advocating for the inclusion of persons with disabilities in areas such as the building of inclusive health care systems to ensure that they are legitimate and feasible [46]. Thus, the design and implementation of inclusive health policies and programs is key, as it could reduce health disparities and ultimately improve health outcomes for persons with disabilities.
